# A Method of Q-Matrix Validation for the Linear Logistic Test Model

**DOI:** 10.3389/fpsyg.2017.00897

**Published:** 2017-05-30

**Authors:** Purya Baghaei, Christine Hohensinn

**Affiliations:** ^1^English Department, Mashhad Branch, Islamic Azad UniversityMashhad, Iran; ^2^Department of Psychology, University of ViennaVienna, Austria

**Keywords:** linear logistic test model, Rasch model, weight matrix, validation

## Abstract

The linear logistic test model (LLTM) is a well-recognized psychometric model for examining the components of difficulty in cognitive tests and validating construct theories. The plausibility of the construct model, summarized in a matrix of weights, known as the Q-matrix or weight matrix, is tested by (1) comparing the fit of LLTM with the fit of the Rasch model (RM) using the likelihood ratio (LR) test and (2) by examining the correlation between the Rasch model item parameters and LLTM reconstructed item parameters. The problem with the LR test is that it is almost always significant and, consequently, LLTM is rejected. The drawback of examining the correlation coefficient is that there is no cut-off value or lower bound for the magnitude of the correlation coefficient. In this article we suggest a simulation method to set a minimum benchmark for the correlation between item parameters from the Rasch model and those reconstructed by the LLTM. If the cognitive model is valid then the correlation coefficient between the RM-based item parameters and the LLTM-reconstructed item parameters derived from the theoretical weight matrix should be greater than those derived from the simulated matrices.

## Background

The linear logistic test model (LLTM; Fischer, 1973) is an extension of the Rasch model (RM, Rasch, 1960/1980) which imposes some linear constraints on the item parameters. LLTM assumes that item difficulty β_*i*_ is a weighted sum of the basic parameters η_*j*_. The item response function for the standard dichotomous Rasch model is expressed as follows:

(1) $$\begin{array}{r} \text{P}\left(X_{\nu i}=1|\theta_{\nu },\beta_{i}\right)=\frac{\text{exp}\left(\theta_{\nu }-\beta_{i}\right)}{1+\text{exp}\left(\theta_{\nu }-\beta_{i}\right)} \end{array}$$

LLTM imposes the following linear constraint on the difficulty parameter β_*i*_:

(2) $$\begin{array}{r} \beta_{i}=\sum_{j}^{p}q_{ij}\eta_{j}+c \end{array}$$

where *q*_*ij*_ is the given weight of the basic parameter *j* on item *i*, η_*j*_ is the estimated difficulty of the basic parameter *j*, and *c* is a normalization constant. The motivation behind this extension of the Rasch model is to investigate and parameterize the cognitive operations and mental processes that are involved in solving the items (basic parameters) (Fischer, 1995)^1^.

Under the LLTM, theoretically, the difficulty parameters of the processes hypothesized to be involved in solving the items η_*j*_ add up and constitute the Rasch model item difficulty parameters β_*i*_. In other words, item difficulty is an additive function of the basic parameters η_*j*_. That is, if the construct theory suggests that two cognitive operations with difficulty parameters of η_1_ and η_2_ are needed to solve item *i* then the Rasch model-based difficulty of item *i* is: β_*i*_ = η_1 +_ η_2_. And if item *j* requires the same two operations plus another operation with difficulty η_3_, then β_*j*_ = η1 + η2 + η3 *and β*_*j*_ − β_*i*_ = η_3_ (Fischer, 1995).

LLTM can also be used in investigating the impact of construct irrelevant factors, such as test method, item position, length of the item text, etc., on item difficulty and the impact of experimental conditions such as training and therapy (for applications of LLTM see Kubinger, 2009; Baghaei and Kubinger, 2015; Hohensinn and Baghaei, 2017).

To estimate LLTM a matrix of weights **Q** which defines the relationship between items and cognitive operations should be specified. The weight matrix or the Q-matrix **Q** contains items' underlying cognitive operations or basic parameters η_*j*_ along with their weights *q*_*ij*_ on each item *i*. The weight matrix is in fact the construct theory under investigation or “the researcher's hypothesis about the factors causing differences of difficulties between items” (Fischer, 2005, p. 509). Misspecification of the weight matrix has profound effects on model fit and parameter estimation (Baker, 1993; Fischer, 2005). Misspecification occurs when researchers fail to take into account the relevant cognitive processes which are involved in answering the items or when the assignment of cognitive processes to items and their weights are wrong.

One common approach to test the validity of the hypothesized theory reflected in the weight matrix is to compare the fit of the LLTM with the fit of the Rasch model. LLTM is a more restricted model than the Rasch model and, therefore, the LLTM and the Rasch model are hierarchically related or are nested models. Likelihood ratio (LR) test can be used to compare the fit of nested models. The deviance of −2 times log-likelihoods of the two models is approximately chi-square distributed with degrees of freedom equal to the difference between the numbers of parameters in the models (Fischer, 1973). Comparing the models entails the ratio of the two likelihoods (Lindsey, 1997; Agresti, 2013):

(3) $$\begin{array}{r} \text{D}=-2log\frac{L_{LLTM}}{L_{RM}} \end{array}$$

A condition for the LR test is that the superior model, in this case the Rasch model, should fit the data (Fischer, 2005)^2^.

The problem with the LR test is that it is almost always significant and, consequently, LLTM is rejected (Fischer and Formann, 1982) and one wonders whether the hypothesized cognitive model is useful in accounting for item difficulties. Fischer and Formann (1982) state that a good fit of the model is difficult to attain or is only attained if a test is deliberately constructed according to a cognitive model. Identifying meaningful cognitive operations for existing tests not developed according to a cognitive model is extremely challenging. Nevertheless, “…such statistical significances ought not to be over-rated, because in many cases relatively large samples of data were used for testing hypotheses about only a few parameters, i.e., the tests were rather powerful; moreover, ultimately any significance criterion is arbitrary” (Fischer and Formann, 1982, p. 412).

For this reason researchers mostly rely on the correlation between difficulty parameters resulted from fitting the Rasch model and those reconstructed by the LLTM. If the weight matrix is specified correctly, i.e., if the construct theory defined in terms of the basic parameters and their weights is valid, the item parameters from the Rasch model should be the same as those reproduced by the LLTM, except for random error. Nevertheless, there is no cut-off value for the magnitude of the correlation coefficient to ascertain the validity of the cognitive model. Correlations between 0.75 (Baghaei and Ravand, 2015) and 0.98 (Sonnleitner, 2008) have been reported in the literature.

### Parallel analysis for weight matrix validation

Horn (1965) suggested parallel analysis (PA) as a technique for deciding on the number of factors to extract in exploratory factor analysis. The logic of PA in factor analysis is that if the extracted factors have any substantive meaning their eigenvalues should be greater than the eigenvalues of simulated data with the same specifications.

Since it is very difficult to attain good fit for the LLTM researchers almost always rely on the correlation between the Rasch model (RM) item parameters and LLTM reconstructed item parameters to evaluate the usefulness of their cognitive theory in accounting for variations in item parameters. However, there is no recommended cut-off value in the literature on how large the correlation should be to confirm the validity of the weight matrix and by implication the explanatory usefulness of the cognitive model postulated. Furthermore, there is no statistical significance test for this correlation. Fischer and Formann (1982) note that “it is meaningless to test such a correlation for significance since the H_0_ that the LLTM holds cannot be expressed by “*r* = 0,” but would have to be “*r* = 1,” for which no adequate test statistics exist” (p. 412).

The empirical validity of the weight matrix is in fact evidence for the validity of the postulated cognitive model (Baghaei and Kubinger, 2015). Based on the logic of PA, we suggest that if the cognitive model is valid then the correlation coefficient between the Rasch model item parameters and the LLTM-reconstructed item parameters derived from the theoretical weight matrix should be greater than the correlation coefficients derived from random simulated weight matrixes with the same number of items and cognitive operations.

In short, we suggest that to evaluate the substantive plausibility of the cognitive model researchers can simulate random weight matrices with the same number of items and operations as there are in the actual theoretical weight matrix and feed them into the LLTM analysis and compute the average of the correlations yielded by these “fake” weight matrixes. We expect the correlation from the theoretical weight matrix to be greater than 95% of these correlations. In this case there is evidence for the usefulness and plausibility of the cognitive model to account for variance in item parameters.

## Methods

### Simulations

To study the appropriateness and the feasibility of our approach we ran simulations based on two empirical data sets to which LLTM had been fitted before. Ghahramanlou et al. (2017) analyzed 23 items in the listening comprehension section of the International English Language Testing System (IELTS) with LLTM. Content analysis of the test by domain experts revealed six processes underlying the test. LLTM analysis of the test had a poorer fit compared to the RM according to likelihood ratio (LR) test. The correlation between the LLTM-reconstructed item parameters and the RM item parameters was *r* = 0.85.

Baghaei and Ravand (2015) analyzed a 20-item high-stakes reading comprehension test in English as a foreign language and derived five cognitive processes underlying the test. The LR test showed that the LLTM analysis of the test with 17 items (three items were deleted to attain fit to the RM) had a poorer fit than the standard Rasch model. The correlation between the LLTM-reconstructed item parameters and the RM-based item parameters was *r* = 0.72. We applied our proposed method to these two datasets to evaluate the explanatory power of the Q-matrices in these two studies. For both real data sets, the information criteria AIC and BIC were calculated which are displayed in Table 1. All analyses of the empirical data sets and the simulations were run with R (R Core Team, 2016)^3^. The weight matrices of both empirical data sets are shown Table A and B in the Appendix.

**Table 1:** Deviance, number of estimated parameters and information criteria AIC and BIC for the RM and the LLTM of the two real data sets.

	**Deviance**	**#Parameters**	**AIC**	**BIC**
RM Listening	4931.68	22	4975.68	5057.81
LLTM Listening	5512.87	6	5524.87	5547.27
RM Reading	5532.41	16	5564.41	5628.24
LLTM Reading	5839.56	5	5849.56	5869.51

For the simulation study, three different scenarios were implemented. In the first two scenarios, weight matrices were intentionally misspecified with the aim of checking the impact on the correlation between the parameters. In Scenario 1, the weight matrix was misspecified to a high degree by simulating the design matrices almost completely at random. In Scenario 2 another approach was taken: the empirical weight matrix was taken as a starting point and perturbations were imposed gradually. Thus, Scenario 1 serves to get the lowest possible benchmark. In practice, a theoretically derived weight matrix should show a better fit and produce a higher correlation than a completely random matrix. Scenario 2 sets a low benchmark (but higher than in Scenario 1). Finally, Scenario 3 serves to get an upper benchmark for the fit of an empirical weight matrix.

### Scenario 1

The dimensions of the original weight matrices **Q** of the empirical data sets were taken and randomized weight matrices were created with the same dimensions. In the empirical data sets, the proportions of 0′s and 1′s in the matrix **Q**_L_ of the listening test were 61.59% and 38.41%, respectively. For the reading test the proportions of 0′s and 1′s in the weight matrix **Q**_R_ were 65.88 and 34.12%, respectively. To create the random weight matrices, each entry of the matrix *q*_*ij*_ was sampled from *q*_*ij*_ ∈ {0;1} and the proportion of 1′s was altered between 30 and 70%. The reason for deciding to limit the proportion of 1′s between 0.3 and 0.7 was that in applications of the LLTM the proportion of 1′s in design matrices of the LLTM is smaller than the proportion of 0′s and rarely exceeds 0.7 (for a typical example of an application of the LLTM see Freund et al., 2008). One-thousand random weight matrices **Q**_S_ were generated and the original empirical data sets were analyzed using these 1,000 matrices. The correlations between the item parameters from the Rasch model of the empirical data with those reconstructed from the LLTM basic parameters using the simulated weight matrices were calculated. In addition the LR test and information criteria AIC and BIC comparing the LLTM with the Rasch model were computed. If the empirical weight matrices **Q**_R_ and **Q**_L_ are substantively valid, we expect the majority of the **Q**_S_′ s lead to correlations lower than those based on actual matrices **Q**_R_and **Q**_L_ because they do not rest on a theoretical rationale but are only randomly generated. Table C in the Appendix shows one of the one thousand simulated weight matrices for the reading comprehension test as an example. As Table C in the Appendix shows, the dimensions of the simulated matrix is equal to those of the empirical matrix (Table B in the Appendix). The 0′s and 1′s are sampled randomly and the proportion of 0′s and 1′s is set to 50 percent in this example matrix.

### Scenario 2

Weight matrices created completely at random, are a rather low benchmark for validity. Therefore, a second scenario was implemented: the new weight matrices were not sampled randomly; instead, the original matrices **Q**_R_ and **Q**_L_ were taken and were modified to introduce some amount of “randomness” or noise to them. For this purpose a varying number of rows (representing the items) of the **Q**_R_ and **Q**_L_ were misplaced.

Misplacing the rows in the listening weight matrix **Q**_L_ allowed for 3.556874e + 14 permutations, while the reading test weight matrix **Q**_R_ allowed 2.585202e + 22 permutations. A random sample of 1,000 permutations were selected as 1,000 new modified weight matrices **Q**_M_, since it was impossible to analyze all the permutations. As in Scenario 1, these matrices were used for an LLTM analysis on the original empirical data sets. The correlations of the item parameters as well as the LR tests and information criteria were computed.

Table D in the Appendix shows as an example of one of the perturbed weight matrices for the reading comprehension test. In this example, only two rows of the empirical weight matrix are switched—the rows for item 1 and item 2 (compare to Table B in the Appendix). Thus, in contrast to Scenario 1, the matrices of Scenario 2 are much more similar to the empirical matrix. The degree of misspecification of the matrices in Scenario 2 are much smaller than that in Scenario 1.

### Scenario 3

Scenarios 1 and 2 aimed to provide a “lower benchmark.” i.e., how high the correlations between item parameters can be in the case of just “randomized” or “partly randomized” weight matrices. Now, with Scenario 3, we wanted to get an impression of the upper benchmark. Therefore we studied how high the correlations could be if the weight matrix is “perfectly” specified.

The starting point was again the original weight matrices **Q**_R_ and **Q**_L__._ Furthermore, the estimated basic parameters of the empirical analyses were taken and the item parameters were reconstructed. Subsequently, 1,000 data sets (with dimensions equal to those of the empirical data sets) were simulated on the basis of these reconstructed item parameters. That is, data sets were generated on the basis of the LLTM which means that they had a “perfect” fit (besides the sampling error) to the given weight matrices. Again, these 1,000 data sets were analyzed using the LLTM with **Q**_R_ and **Q**_L_. The correlations between item parameter, the LR tests, AIC, and BIC were computed. The estimation method for the Rasch models and LLTM was conditional maximum likelihood method.

## Results

### Scenario 1

As mentioned in the previous section, two empirical examples were chosen for which LLTM analyses had already been conducted. The results in both cases showed a significant LR test. The correlation between the RM item parameters and the item parameters reconstructed by the LLTM was *r* = 0.8506 for the listening test and *r* = 0.7208 for the reading test.

The proportion of 0′s and 1′s in the weight matrices were varied from 30 to 70% 1′s in the simulations. The descriptive statistics for the correlations between the item parameters from the Rasch model and the LLTM are shown in Table 2. As the table shows, the proportion of 0′s and 1′s had no impact on the correlations of item parameters. Findings revealed that for the listening test the empirical correlation of the original weight matrix (*r* = 0.85) is within the upper 5% of the correlations obtained from the simulated matrices. For the reading test, the empirical correlation (*r* = 0.72) is just below the 95% percentile of the randomized weight matrices. This casts some doubt on the validity of the empirical weight matrix in explaining the cognitive processes underlying the reading test. Table 2 shows that the mean and the median of the correlations for random Q-matrices are between 0.50 and 0.55. That is, a correlation coefficient as high as 0.55 is expected between RM item parameters and LLTM- reconstructed parameters even when the weight matrix is developed haphazardly without any substantive theory. Therefore, a valid weight matrix should yield a substantially higher correlation than 0.55 to be considered meaningful from a theoretical point of view.

**Table 2:** Descriptive statistics for the correlations obtained from simulated weight matrices.

	**Min**	**Percentil 5%**	**1st Quartile**	**Median**	**Mean**	**3rd Quartile**	**Percentile 95%**	**Max**
Listening 30	0.1503	0.2892	0.4142	0.5031	0.5013	0.5890	0.7111	0.8690
Listening 40	0.1532	0.2905	0.4158	0.5075	0.5020	0.5921	0.7073	0.8819
Listening 50	0.1506	0.2960	0.4420	0.5176	0.5117	0.6054	0.7030	0.8870
Listening 60	0.0647	0.2949	0.4107	0.5037	0.5016	0.5880	0.7096	0.8252
Listening 70	0.1255	0.2891	0.4161	0.5036	0.5042	0.5925	0.7071	0.8377
Reading 30	0.1100	0.3098	0.4403	0.5507	0.5448	0.6495	0.7670	0.8955
Reading 40	0.1275	0.2879	0.4355	0.5409	0.5386	0.6467	0.7696	0.8965
Reading 50	0.1183	0.2823	0.4288	0.5434	0.5350	0.6375	0.7874	0.9292
Reading 60	0.1400	0.2882	0.4368	0.5448	0.5367	0.6329	0.7606	0.9105
Reading 70	0.0716	0.2762	0.4383	0.5337	0.5347	0.6417	0.7674	0.8919

*The number in the row name refers to the proportion of 0′s and 1′s, thus Listening 30 means, that the weight matrices were simulated with a proportion of 30:70 for 0′s and 1′s*.

Besides the correlations, the LR test comparing the likelihood of the LLTM to that of the Rasch model was performed for each of the randomized weight matrices. As expected, all LR tests were significant (*p* < 0.05) indicating a worse fit for the LLTM compared to the Rasch model. In addition, the information criteria AIC and BIC were calculated for the LLTM of the simulated weight matrices and the Rasch model. Descriptive statistics as well as the percentage of values that favored the LLTM are provided in Table 3. In accordance to the results of the LR test, for each simulated weight matrix, the Rasch model had a lower AIC/BIC compared to the LLTM suggesting a worse fit for the LLTM compared to the Rasch model. Note that two out of the 1,000 randomized generated weight matrices (with 60% 1′s) for the listening weight matrix did not lead to a convergent solution. For the reading test weight matrices, 18 out of the 1,000 simulated weight matrices with 30% 1′s did not converge. The results are based only on those matrices with convergent solutions.

**Table 3:** Descriptive statistics of the information criteria AIC and BIC for the LLTM analysis based on the simulated weight matrices.

	**AIC**				**BIC**			
	**Min**	**Mean**	**Max**	**Percent**	**Min**	**Mean**	**Max**	**Percent**
Listening 30	5,456	6,399	6,829	0	5,478	6,421	6,852	0
Listening 40	5,432	6,400	6,836	0	5,454	6,423	6,859	0
Listening 50	5,418	6,383	6,827	0	5,441	6,406	6,850	0
Listening 60	5,628	6,400	6,866	0	5,651	6,422	6,888	0
Listening 70	5,557	6,397	6,849	0	5,580	6,419	6,871	0
Reading 30	5,673	5,976	6,169	0	5,693	5,996	6,189	0
Reading 40	5,674	5,979	6,162	0	5,694	5,999	6,182	0
Reading 50	5,686	5,986	6,161	0	5,706	6,006	6,181	0
Reading 60	5,668	6,002	6,182	0	5,668	6,002	6,182	0
Reading 70	5,679	5,982	6,170	0	5,699	6,002	6,190	0

*Percent is the percentage of AIC/BIC values of the LLTM that are smaller than the AIC/BIC value of the Rasch model of the empirical data set*.

### Scenario 2

For Scenario 2, the rows of the empirical weight matrices **Q**_L_ and **Q**_R_ were misplaced. The results are presented for the different amounts of perturbations imposed. Three conditions of small, medium, and large perturbations, depending on the number of rows misplaced, were simulated. Small perturbation means that the new weight matrix is very similar to the original one whereas a high level of perturbation means that the weight matrix is much more similar to a completely randomized weight matrix similar to Scenario 1. For the listening test with 23 rows, displacement of 2 to 7 rows were considered as “small,” 8 to 15 as “medium” and 16 to 23 as “large.” For the reading test with 17 rows, displacement of 2 to 6 rows were considered as “small,” 7 to 12 as “medium,” and 13 to 17 as “large.” The item parameter correlations of Scenario 2 are shown in Table 4. The correlation of item parameters gets lower with a higher number of misplaced rows. Compared to the results of Scenario 1, the range of the correlations is smaller. As in Scenario 1, the LR tests were significant and indicated that the RM fits better than the LLTM. The information criteria again confirm the results of the LR test (see Table 5).

**Table 4:** Descriptive statistics for the correlations obtained from the perturbed weight matrices.

	***n***	**Min**	**Percentile 5%**	**1st Quartile**	**Median**	**Mean**	**3rd Quartile**	**Percentile 95%**	**Max**
Listening small	91	0.6610	0.7572	0.7780	0.8337	0.8177	0.8487	0.8626	0.8734
Listening medium	336	0.3414	0.3814	0.4906	0.5649	0.5790	0.6746	0.7604	0.8138
Listening large	573	0.3414	0.3630	0.4229	0.4942	0.4805	0.5276	0.5744	0.6359
Reading small	137	0.5239	0.5360	0.5853	0.6570	0.6529	0.7111	0.7681	0.7966
Reading medium	419	0.2956	0.4547	0.5327	0.5837	0.5880	0.6382	0.7398	0.8195
Reading large	444	0.1634	0.3117	0.3800	0.4493	0.4470	0.5171	0.5926	0.6480

*n is the number of matrices in each category, small, medium, and large*.

**Table 5:** Descriptive statistics for AIC and BIC for the LLTM analysis based on the simulated weight matrices.

	**AIC**				**BIC**			
	**Min**	**Mean**	**Max**	**Percent**	**Min**	**Mean**	**Max**	**Percent**
Listening small	5,461	5,722	6,172	0	5,483	5,744	6,195	0
Listening medium	5,772	6,328	6,659	0	5,795	6,350	6,681	0
Listening large	6,146	6,407	6,650	0	6,168	6,429	6,672	0
Reading small	5,779	5,907	6,009	0	5,799	5,927	6,029	0
Reading medium	5,753	5,959	6,120	0	5,773	5,979	6,140	0
Reading large	5,921	6,049	6,158	0	5,941	6,068	6,178	0

*Percent is the percentage of AIC/BIC values of the LLTM that are smaller than the AIC/BIC value of the Rasch model of the empirical data set*.

### Scenario 3

For the LLTM-fitting data sets generated on the basis of the empirical weight matrices, the results of the item parameter correlations are displayed in Table 6.

**Table 6:** Descriptive statistics for the correlations for the simulated LLTM-fitting data sets based on the theoretical weight matrices **Q**_L_and **Q**_R_.

	**Min**	**Percentile 5%**	**1st Quartile**	**Median**	**Mean**	**3rd Quartile**	**Percentile 95%**	**Max**
Listening	0.9875	0.9917	0.9938	0.9951	0.9949	0.9962	0.9975	0.9984
Reading	0.9472	0.9736	0.9815	0.9860	0.9851	0.9898	0.9939	0.9972

Table 6 indicates that when the LLTM perfectly fits we expect the correlation between RM item parameters and LLTM-reconstructed parameters to be greater than *r* = *0*.95. This scenario of simulations sets an upper bound for the expected correlation. Note that such a high magnitude of correlation is rarely obtained in practice as empirical data never perfectly fit mathematical models. For the listening data 4.7% of the LR tests and for the reading data 4.5% of the LR tests were significant, indicating that the RM fits better than LLTM. This finding was expected—the data were generated using the empirical weight matrix to have a “perfect fit” (besides the random error) to LLTM. Thus, the number of significant LR tests reflects the type-I-risk which was set at 5% and is, therefore, satisfactorily approximated.

In addition, information criteria AIC and BIC were calculated for each of the simulated data sets. Because, the comparison of AICs and BICs for model selection is only possible for identical data sets, AICs and BICs of the Rasch model and the LLTM were compared for each data set separately. That means, for each data set, the Rasch model and the LLTM were estimated and the AICs and BICs were compared. For the data simulation that was based on the empirical weight matrix or the Listening data, the AIC favored the LLTM in 6 of 1,000 data sets, the BIC always preferred the RM. For the six data sets, where AIC favored the LLTM, the difference between the AICs was very small (with a maximum difference of 4.64). For the data sets that were simulated according to the empirical weight matrices of the Reading test very similar results were found: the BIC of the LLTM was never smaller than that of the Rasch model - that is, the BIC always preferred the Rasch model. According to the AIC, in 22 of 1,000 data sets, the AIC was smaller for the LLTM than for the Rasch model with a maximum difference of 12.47 between the two AIC values.

## Conclusions

In this article a method for evaluating the weight matrix in the linear logistic test model was proposed. The method is based on parallel analysis suggested by Horn (1965) for deciding on the number of factors to extract in exploratory factor analysis. Our method rests on the argument that if the weight matrix is substantively valid, i.e., the cognitive operations underlying the test are correctly specified, the correlation coefficient between the RM item parameters and LLTM-reconstructed item parameters using the empirical weight matrix should be higher than the correlations yielded by using random simulated weight matrices. It is important to note that the procedure outlined here is not evidence for the fit of the LLTM but evidence for the relative usefulness of the cognitive model postulated. We showed with two empirical examples how the method works. An R package was also presented to perform our proposed method for any other LLTM analysis. The simulation results can also be used to set benchmarks for minimum and maximum correlations to expect between RM item parameters and LLTM reconstructed item parameters in practice as evidence for the validity of the substantive theory underlying a test. Simulations showed that when the weight matrix is generated completely at random a coefficient of correlation of 0.50 is obtained. Therefore, for a weight matrix to be meaningful it should produce a correlation coefficient way above 0.50. The 95% percentile of the distribution of correlations in scenario 1 for different proportion of 1′s and 0′s was between 0.70 and 0.78. Therefore, a correlation coefficient of 0.78 can be set as a minimum cut-off value for meaningful weight matrix.

## Author contributions

PB developed the idea and wrote the theoretical part of the manuscript. CH performed the simulation studies.

### Conflict of interest statement

The authors declare that the research was conducted in the absence of any commercial or financial relationships that could be construed as a potential conflict of interest.

## Footnotes

^1^IRT models, in general, are not designed to reflect the cognitive processes which underlie the responses. These models are commonly based on mathematical or measurement properties such as sufficiency, separation, and additively and are not linked with the item response generation mechanisms (van der Mass et al., 2011). According to van der Mass et al. (2011) there is a paucity of such models that can be problematic as validity evidence requires models that address the information processing theories of tests (see Baghaei and Tabatabaee-Yazdi, 2016). Other psychometric models that addresses the information processing theories of the item responses are the class of cognitive diagnostic models (de la Torre, 2009), diffusion model, and the race model (Tuerlinckx and De Boeck, 2005).

^2^For the Likelihood-Ratio test formula, the typical notation in the LLTM literature is used here (see Fischer, 1973).

^3^To perform our method we have written an R package that can be downloaded under http://github.com/christinehohensinn/parAL.
