# Supplementary material for: A Method of Q-Matrix Validation for the Linear Logistic Test Model
Source: Front Psychol. 2017 May 30;8:897. doi: 10.3389/fpsyg.2017.00897 (PMC5448235; doi:10.3389/fpsyg.2017.00897)
Supplement: Supplementary file 1 [file DataSheet1.docx]

**Appendix**

Table A: Empirical weight matrix for the listening comprehension test. B denotes basic parameters.

|  | B1 | | B2 | | B3 | B4 | B5 | B6 |
| --- | --- | --- | --- | --- | --- | --- | --- | --- |
| Item 1 | 0 | 0 | | 1 | | 0 | 0 | 0 |
| Item 2 | 0 | 0 | | 1 | | 0 | 0 | 0 |
| Item 3 | 1 | 1 | | 0 | | 0 | 0 | 0 |
| Item 4 | 0 | 1 | | 1 | | 0 | 0 | 0 |
| Item 5 | 0 | 1 | | 1 | | 0 | 0 | 0 |
| Item 6 | 0 | 1 | | 0 | | 0 | 0 | 0 |
| Item 7 | 0 | 0 | | 1 | | 0 | 1 | 0 |
| Item 8 | 0 | 0 | | 1 | | 0 | 1 | 0 |
| Item 9 | 0 | 1 | | 1 | | 1 | 1 | 0 |
| Item 10 | 0 | 1 | | 1 | | 0 | 0 | 1 |
| Item 11 | 0 | 1 | | 0 | | 0 | 1 | 0 |
| Item 12 | 0 | 1 | | 1 | | 1 | 1 | 0 |
| Item 13 | 0 | 0 | | 1 | | 0 | 0 | 0 |
| Item 14 | 0 | 0 | | 1 | | 1 | 1 | 0 |
| Item 15 | 0 | 0 | | 1 | | 1 | 1 | 0 |
| Item 16 | 0 | 1 | | 1 | | 0 | 0 | 0 |
| Item 17 | 1 | 0 | | 1 | | 0 | 0 | 0 |
| Item 18 | 0 | 0 | | 1 | | 0 | 0 | 0 |
| Item 19 | 0 | 1 | | 1 | | 0 | 1 | 0 |
| Item 20 | 0 | 0 | | 1 | | 0 | 0 | 0 |
| Item 21 | 1 | 1 | | 1 | | 1 | 1 | 0 |
| Item 22 | 0 | 0 | | 1 | | 1 | 0 | 1 |
| Item 23 | 0 | 0 | | 1 | | 1 | 1 | 0 |

Table B: Empirical weight matrix for the Reading comprehension test. B denotes basic parameters.

|  | B1 | | B2 | | B3 | B4 | B5 |
| --- | --- | --- | --- | --- | --- | --- | --- |
| Item 1 | 0 | 1 | | 0 | | 0 | 1 |
| Item 2 | 1 | 0 | | 0 | | 0 | 1 |
| Item 3 | 0 | 1 | | 0 | | 0 | 1 |
| Item 4 | 0 | 0 | | 1 | | 1 | 1 |
| Item 5 | 0 | 0 | | 0 | | 0 | 1 |
| Item 6 | 0 | 0 | | 0 | | 1 | 1 |
| Item 7 | 0 | 0 | | 0 | | 1 | 1 |
| Item 8 | 0 | 1 | | 0 | | 0 | 1 |
| Item 9 | 1 | 0 | | 0 | | 0 | 0 |
| Item 10 | 1 | 1 | | 0 | | 0 | 1 |
| Item 11 | 0 | 0 | | 0 | | 1 | 1 |
| Item 12 | 0 | 0 | | 1 | | 0 | 0 |
| Item 13 | 0 | 1 | | 0 | | 0 | 0 |
| Item 14 | 0 | 1 | | 0 | | 0 | 0 |
| Item 15 | 0 | 1 | | 0 | | 0 | 0 |
| Item 16 | 1 | 0 | | 0 | | 0 | 0 |
| Item 17 | 0 | 0 | | 0 | | 1 | 1 |

Table C: A perturbed weight matrix for the reading comprehension test. B denotes basic parameters.

|  | B1 | | B2 | | B3 | B4 | B5 |
| --- | --- | --- | --- | --- | --- | --- | --- |
| Item 1 | 1 | 1 | | 0 | | 1 | 0 |
| Item 2 | 1 | 1 | | 1 | | 0 | 1 |
| Item 3 | 1 | 1 | | 0 | | 0 | 0 |
| Item 4 | 0 | 0 | | 1 | | 1 | 0 |
| Item 5 | 1 | 1 | | 1 | | 1 | 1 |
| Item 6 | 0 | 0 | | 1 | | 1 | 0 |
| Item 7 | 0 | 0 | | 0 | | 1 | 0 |
| Item 8 | 1 | 1 | | 1 | | 1 | 1 |
| Item 9 | 0 | 1 | | 0 | | 1 | 0 |
| Item 10 | 1 | 0 | | 1 | | 1 | 0 |
| Item 11 | 1 | 0 | | 0 | | 0 | 0 |
| Item 12 | 0 | 1 | | 0 | | 0 | 1 |
| Item 13 | 0 | 0 | | 1 | | 0 | 1 |
| Item 14 | 1 | 1 | | 1 | | 0 | 1 |
| Item 15 | 0 | 1 | | 1 | | 0 | 1 |
| Item 16 | 0 | 0 | | 0 | | 1 | 0 |
| Item 17 | 1 | 1 | | 1 | | 0 | 1 |

Table D: A perturbed weight matrix for the reading comprehension test. B denotes basic parameters.

|  | B1 | | B2 | | B3 | B4 | B5 |
| --- | --- | --- | --- | --- | --- | --- | --- |
| Item 1 | 1 | 0 | | 0 | | 0 | 1 |
| Item 2 | 0 | 1 | | 0 | | 0 | 1 |
| Item 3 | 0 | 1 | | 0 | | 0 | 1 |
| Item 4 | 0 | 0 | | 1 | | 1 | 1 |
| Item 5 | 0 | 0 | | 0 | | 0 | 1 |
| Item 6 | 0 | 0 | | 0 | | 1 | 1 |
| Item 7 | 0 | 0 | | 0 | | 1 | 1 |
| Item 8 | 0 | 1 | | 0 | | 0 | 1 |
| Item 9 | 1 | 0 | | 0 | | 0 | 0 |
| Item 10 | 1 | 1 | | 0 | | 0 | 1 |
| Item 11 | 0 | 0 | | 0 | | 1 | 1 |
| Item 12 | 0 | 0 | | 1 | | 0 | 0 |
| Item 13 | 0 | 1 | | 0 | | 0 | 0 |
| Item 14 | 0 | 1 | | 0 | | 0 | 0 |
| Item 15 | 0 | 1 | | 0 | | 0 | 0 |
| Item 16 | 1 | 0 | | 0 | | 0 | 0 |
| Item 17 | 0 | 0 | | 0 | | 1 | 1 |
